# Supplementary material for: The Dual Role of Necroptosis in Pancreatic Ductal Adenocarcinoma
Source: Int J Mol Sci. 2023 Aug 10;24(16):12633. doi: 10.3390/ijms241612633 (PMC10454309; doi:10.3390/ijms241612633)
Supplement: Supplementary file 1 [file ijms-24-12633-s001.zip › ijms-2523522-supplementary.pdf]

Supplementary: Table S1. Malignant variants of PDAC [1].

PDAC variant	Histologic hallmarks	Non-canonical genetic Signatures
**Adenosquamous Carcinoma**	Both adenomatous and squamous cells	UPF1[2], MHL1, MHL2, PMS2, MSH6[3]
**Undifferentiated Carcinoma**	Necrotic areas surrounded by a wall of packed epithelial cells without abundant desmoplastic stroma	SERPINIA3, MAGEB4, GLI3, MEGF8, TTN[4],BRCA2[5](UCOGC) SMARCB1[6](rhabdoid variant)
**Micropapillary Carcinoma**	Tight and small neoplastic cell clusters, surrounded by a peculiar cleft	N.a.
**Signet-ring cell Carcinoma**	Cells with vacuolated cytoplasm and peripheric irregular nuclei	N.a.
**Large-duct type Carcinoma**	Large ductal infiltrative elements and cells with abundant microvesicular cytoplasm	N.a
**Colloid Carcinoma**	Pools of extracellular mucin with neoplastic ductal epithelial cells	GNAS [7]ATM [8], MHL1, MHL2, PMS2, MSH6[9]
**Medullary carcinoma**	Syncytial growth patterns that expand tumor borders, extensive necrosis	MHL1, MHL2, PMS2, MSH6 [10],[11] POLE [12]
**Hepatoid carcinoma**	Cords of polygonal cells with eosinophilic cytoplasm	BAP1, Notch [13]

*

UCOGC (Undifferentiated carcinoma with osteoclast-like giant cells).

Supplementary: Table S2. PDAC precursor lesions [1].

PDAC precursor	Features	Molecular landscape
**Pancreatic intraepithelial neoplasia (PanIN)**<br>- low-grade (LG)<br>- high-grade (HG)	Microscopic (<5 mm in diameter) lesions arising in small pancreatic ducts [14]	KRAS hotspot mutations (>90% prevalence);<br>CDKN2A/p16, TP53, SMAD4 (rare) loss (HG);<br>telomere shortening (>90% prevalence) [14, 15, 16, 17,18]
**Intraductal papillary mucinous neoplasm (IPMN)**<br>- low-grade (LG)<br>- high-grade (HG)<br>- with associated invasive carcinoma (IPMN-AIC)	Macroscopic (>1cm in diameter), proliferations of mucinous cells forming papillary projections within the main pancreatic duct or its major branches; gastric, intestinal and pancreatobiliary type) [18, 19 ,20]	KRAS hotspot mutations (40-80% prevalence);<br>GNAS mutations 40-70% prevalence); *RNF4 alterations;*<br>CDKN2A/p16 loss (HG>LG)<br>TP53 mutations (HG>LG);<br>PIK3CA, BRAF, PTEN and STK11 alterations (low prevalence) [14, 21, 22, 23]
**Mucinous cystic neoplasm (MCN)**<br>- with low-grade (LG) dysplasia<br>- with high-grade (HG) dysplasia<br>- with associated invasive carcinoma (MCN-AIC), rare (15%)	Large cysts with single-layer mucin-producing epithelium and distinctive ovarian-like stroma No connection to the duct system. [24,25,26]	KRAS hotspot mutations;<br>*RNF4 alterations;*,<br>TP53, CDKN2A/p16 , SMAD4, TGFBR2 (HG and MCN-AIC);<br>PIK3CA (rare, HG).<br>No *GNAS* mutations [14,27,28]
**Intraductal tubulopapillary neoplasm (ITPN)**	Solid mass within dilated pancreatic ducts, composed of back-to-back tubular glands with high-grade dysplasia and ductal differentiation and no mucin secretion [29, 30]	*MCL-1* amplification.<br>*FGFR2* fusions.<br>Alterations of *PIK3CA*, *PIK3CB*, *INPP4A*, *PTEN* (pI3K pathway*)*;<br>*MLL1*, *MLL2*, *MLL3*, *BAP1*, *PBRM1*, *EED*, *ATRX* (chromatin remodeling) [31, 30]
**Intraductal oncocytic papillary neoplasm (IOPN)**	Intraductal solid nodules or papillary projections lined by mitochondria-rich oncocytic cells, within dilated pancreatic ducts [32]	*PRKA*CB and *PRKACA* fusions.<br>ARHGAP26, ASXL1, EPHA8, ERBB4 mutations.<br>No *KRAS* mutation<br>No *GNAS* mutations [33,34]

Supplementary References:

1. Nagtegaal, ID.; Odze, RD.; Klimstra, D.; Paradis, V.; Rugge, M.; Schirmacher, P.; Washington, KM.; Carneiro F.; Cree, IA. WHO Classification of Tumours Editorial Board. The 2019 WHO classification of tumours of the digestive system. *Histopathology.* **2020**;76(2):182-188. doi: 10.1111/his.13975.
2. Luchini, C.: Pea, A.; Lionheart, G.; Mafficini, A.; Nottegar, A.; Veronese, N.; Chianchiano, P.; Brosens, LA.; Noë, M.; Offerhaus, GJA.; Yonescu, R.; Ning, Y.; Malleo, G.; Riva, G.; Piccoli, P.; Cataldo, I.; Capelli, P.; Zamboni, G.; Scarpa, A.; Wood, LD. Pancreatic undifferentiated carcinoma with osteoclast-like giant cells is genetically similar to, but clinically distinct from, conventional ductal adenocarcinoma. *J Pathol*. **2017**;243(2):148-154. doi: 10.1002/path.4941.
3. Yang, G.; Yin, J.; Ou, K.; Du, Q.; Ren, W.; Jin, Y.; Peng, L.; Yang, L. Undifferentiated carcinoma with osteoclast-like giant cells of the pancreas harboring KRAS and BRCA mutations: case report and whole exome sequencing analysis. BMC *Gastroenterol.* **2020**;20(1):202. doi: 10.1186/s12876-020-01351-7.
4. Agaimy, A.; Haller, F.; Frohnauer, J.; Schaefer, IM.; Ströbel, P.; Hartmann, A.; Stoehr, R.; Klöppel, G. Pancreatic undifferentiated rhabdoid carcinoma: KRAS alterations and SMARCB1 expression status define two subtypes. *Mod Pathol.* **2015**; 28(2):248-60. doi: 10.1038/modpathol.2014.100.
5. Yamada, M.; Sekine, S.; Ogawa, R.; Taniguchi, H.; Kushima, R.; Tsuda, H.; Kanai, Y. Frequent activating GNAS mutations in villous adenoma of the colorectum. *J Pathol.* **2012**; 228(1):113-8. doi: 10.1002/path.4012.
6. Hutchings, D.; Jiang, Z.; Skaro, M.; Weiss, MJ.; Wolfgang, CL.; Makary, MA.; He, J.; Cameron, JL.; Zheng, L.; Klimstra, DS.; Brand, RE.; Singhi, AD.; Goggins, M.; Klein, AP.; Roberts, NJ.; Hruban, RH. Histomorphology of pancreatic cancer in patients with inherited ATM serine/threonine kinase pathogenic variants. *Mod Pathol*. **2019**;32(12):1806-1813. doi: 10.1038/s41379-019-0317-6.
7. Lupinacci, RM.; Goloudina, A.; Buhard, O.; Bachet, JB.; Maréchal, R.; Demetter, P.; Cros, J.; Bardier-Dupas, A.; Collura, A.; Cervera, P.; Scriva, A.; Dumont, S.; Hammel, P.; Sauvanet, A.; Louvet, C.; Delpéro, JR.; Paye, F.; Vaillant, JC.; André, T.; Closset, J.; Emile, JF.; Van Laethem, JL.; Jonchère, V.; Abd Alsamad, I.; Antoine, M.; Rodenas, A.; Fléjou, JF.; Dusetti, N.; Iovanna, J.; Duval, A.; Svrcek, M. Prevalence of Microsatellite Instability in Intraductal Papillary Mucinous Neoplasms of the Pancreas. *Gastroenterology*. **2018**;154(4):1061-1065. doi: 10.1053/j.gastro.2017.11.009.
8. Kondo, E,; Furukawa, T.; Yoshinaga, K.; Kijima, H.; Semba, S.; Yatsuoka, T.; Yokoyama, T.; Fukushige, S.; Horii, A. Not hMSH2 but hMLH1 is frequently silenced by hypermethylation in endometrial cancer but rarely silenced in pancreatic cancer with microsatellite instability. *Int J Oncol*. **2000**;17(3):535-41. doi: 10.3892/ijo.17.3.535.
9. Banville, N.; Geraghty, R.; Fox, E.; Leahy, DT.; Green, A.; Keegan, D.; Geoghegan, J.; O'Donoghue, D.; Hyland, J.; Sheahan, K. Medullary carcinoma of the pancreas in a man with hereditary nonpolyposis colorectal cancer due to a mutation of the MSH2 mismatch repair gene. *Hum Pathol*. **2006**;37(11):1498-502. doi: 10.1016/j.humpath.2006.06.024.
10. Kryklyva, V.; Ter, Linden E.; Kroeze, LI.; de Over, RM.; van der Kolk, BM.; Stommel, MWJ.; Hermans, JJ.; Luchini, C.; Wood, D.; Hruban, RH.; Nagtegaal, ID.; Ligtenberg, MJL.; Brosens, LAA. Medullary Pancreatic Carcinoma Due to Somatic POLE Mutation: A Distinctive Pancreatic Carcinoma With Marked Long-Term Survival. *Pancreas*. **2020;**49(7):999-1003. doi: 10.1097/MPA.0000000000001588.
11. Chang, JM.; Katariya, NN.; Lam-Himlin, DM.; Haakinson, DJ.; Ramanathan, RK.; Halfdanarson, TR.; Borad, MJ.; Pannala, R.; Faigel, D.; Moss, AA.; Mathur, AK. Hepatoid Carcinoma of the Pancreas: Case Report, Next-Generation Tumor Profiling, and Literature Review. *Case Rep Gastroenterol*. **2016**;10(3):605-612. doi: 10.1159/000448064.
12. Fischer, CG:; Wood LD. From somatic mutation to early detection: insights from molecular characterization of pancreatic cancer precursor lesions. *J Pathol*. **2018** Dec;246(4):395-404. doi: 10.1002/path.5154.
13. Distler, M.; Aust, D.; Weitz, J.; Pilarsky, C.; Grützmann, R. Precursor lesions for sporadic pancreatic cancer: PanIN, IPMN, and MCN. *Biomed Res Int*. **2014**;2014:474905. doi: 10.1155/2014/474905.
14. Singh, K.; Pruski, M.; Bland, R.; Younes, M.; Guha, S.; Thosani, N.; Maitra, A.; Cash, BD.; McAllister, F.; Logsdon, CD.; Chang, JT.; Bailey-Lundberg, JM. Kras mutation rate precisely orchestrates ductal derived pancreatic intraepithelial neoplasia and pancreatic cancer. Lab Invest. 2021 Feb;101(2):177-192*et al. Kras* mutation rate precisely orchestrates ductal derived pancreatic intraepithelial neoplasia and pancreatic cancer. *Lab Invest*. **2021**; 101, 177–192. doi: 10.1038/s41374-020-00490-5
15. Feldmann, G.; Beaty, R.; Hruban, RH.; Maitra, A. Molecular genetics of pancreatic intraepithelial neoplasia. *J Hepatobiliary Pancreat Surg*. **2007**;14(3):224-32. doi: 10.1007/s00534-006-1166-5.
16. Distler, M.; Kersting, S.; Niedergethmann, M.; Aust, DE.; Franz, M.; Rückert, F.; Ehehalt, F.; Pilarsky, C.; Post, S.; Saeger, HD.; Grützmann, R. Pathohistological subtype predicts survival in patients with intraductal papillary mucinous neoplasm (IPMN) of the pancreas. *Ann Surg.* **2013** Aug;258(2):324-30. doi: 10.1097/SLA.0b013e318287ab73.
17. Sadakari, Y.; Ohuchida, K.; Nakata, K.; Ohtsuka, T.; Aishima, S.; Takahata, S.; Nakamura, M.; Mizumoto, K.; Tanaka, M. Invasive carcinoma derived from the nonintestinal type intraductal papillary mucinous neoplasm of the pancreas has a poorer prognosis than that derived from the intestinal type. *Surgery,* **2010** Jun;147(6):812-7. doi: 10.1016/j.surg.2009.11.011.
18. Adsay, NV.; Merati, K.; Basturk, O.; Iacobuzio-Donahue, C.; Levi, E.; Cheng, JD.; Sarkar, FH.; Hruban, RH.; Klimstra, DS. Pathologically and biologically distinct types of epithelium in intraductal papillary mucinous neoplasms: delineation of an "intestinal" pathway of carcinogenesis in the pancreas. *Am J Surg Pathol.* **2004** Jul;28(7):839-48. doi: 10.1097/00000478-200407000-00001.

19. Wu, J.; Matthaei, H.; Maitra, A.; Dal Molin, M.; Wood, LD.; Eshleman, JR.; Goggins, M.; Canto, MI.; Schulick, RD.; Edil, BH.; Wolfgang, CL.; Klein, AP; Diaz, LA Jr.; Allen, PJ.; Schmidt, CM.; Kinzler, KW.; Papadopoulos, N.; Hruban, RH.; Vogelstein, B. Recurrent GNAS mutations define an unexpected pathway for pancreatic cyst development. *Sci Transl Med*. **2011** Jul 20;3(92):92ra66. doi: 10.1126/scitranslmed.3002543.
20. Furukawa, T.; Kuboki, Y.; Tanji, E.; Yoshida, S.; Hatori, T.; Yamamoto, M.; Shibata, N.; Shimizu, K.; Kamatani, N.; Shiratori, K. Whole-exome sequencing uncovers frequent GNAS mutations in intraductal papillary mucinous neoplasms of the pancreas. *Sci Rep*. **2011**;1:161. doi: 10.1038/srep00161.
21. Siddiqui, AA.; Kowalski, TE.; Kedika, R.; Roy, A.; Loren, DE.; Ellsworth, E.; Adler, D.; Finkelstein, SD. EUS-guided pancreatic fluid aspiration for DNA analysis of KRAS and GNAS mutations for the evaluation of pancreatic cystic neoplasia: a pilot study. *Gastrointest Endosc.* **2013** Apr;77(4):669-70. doi: 10.1016/j.gie.2012.11.009.
22. Xie, W.; Liang, H.; Guo, Y.; Xiao, Shu-Yuana, D.Update on mucinous cystic neoplasm of the pancreas: a narrative review. *Journal of Pancreatology*. **2021**; 4(3):p 115-121. doi: 10.1097/JP9.0000000000000074
23. Baker, ML.; Seeley, ES.; Pai, R.; Suriawinata, AA.; Mino-Kenudson, M.; Zamboni, G.; Klöppel, G.; Longnecker, DS. Invasive mucinous cystic neoplasms of the pancreas. *Exp Mol Pathol.* **2012** Dec;93(3):345-9. doi: 10.1016/j.yexmp.2012.07.005.
24. Wilentz, RE.; Albores-Saavedra, J.; Hruban, RH. Mucinous cystic neoplasms of the pancreas. *Semin Diagn Pathol.* **2000**;17:31–42
25. Noë M.; Niknafs N.; Fischer CG.; Hackeng WM.; Beleva Guthrie V.; Hosoda W.; Debeljak M.; Papp E.; Adleff V.; White JR.; Luchini C.; Pea A.; Scarpa A.; Butturini G.; Zamboni G.; Castelli P.; Hong SM.; Yachida S.; Hiraoka N.; Gill AJ.; Samra JS.; Offerhaus GJA.; Hoorens A.; Verheij J.; Jansen C.; Adsay NV.; Jiang W.; Winter J.; Albores-Saavedra J.; Terris B.; Thompson ED.; Roberts NJ.; Hruban RH.; Karchin R.; Scharpf RB.; Brosens LAA.; Velculescu VE.; Wood LD. Genomic characterization of malignant progression in neoplastic pancreatic cysts. *Nat Commun*. **2020**;11(1):4085. doi: 10.1038/s41467-020-17917-8.
26. Wu, J.; Jiao, Y.; Dal Molin, M.; Maitra, A.; de Wilde, RF.; Wood, LD.; Eshleman, JR.; Goggins, MG.; Wolfgang, CL.; Canto, MI.; Schulick, RD.; Edil, BH.; Choti, MA.; Adsay, V.; Klimstra, DS.; Offerhaus, GJ.; Klein, AP.; Kopelovich, L.; Carter, H.; Karchin, R.; Allen, PJ.; Schmidt, CM.; Naito, Y.; Diaz, LA Jr; Kinzler, KW.; Papadopoulos, N.; Hruban, RH.; Vogelstein, B. Whole-exome sequencing of neoplastic cysts of the pancreas reveals recurrent mutations in components of ubiquitin-dependent pathways. *Proc Natl Acad Sci U S A.* **2011**; 27;108(52):21188-93. doi: 10.1073/pnas.1118046108.
27. Yamaguchi, H.; Shimizu, M.; Ban, S.; Koyama, I.; Hatori, T.; Fujita, I.; Yamamoto, M.; Kawamura, S.; Kobayashi, M.; Ishida, K.; Morikawa, T.; Motoi, F.; Unno, M.; Kanno, A.; Satoh, K.; Shimosegawa, T.; Orikasa, H.; Watanabe, T.; Nishimura, K.; Ebihara, Y.; Koike, N.; Furukawa, T. Intraductal tubulopapillary neoplasms of the pancreas distinct from pancreatic intraepithelial neoplasia and intraductal papillary mucinous neoplasms. *Am J Surg Pathol.* **2009**;33(8):1164-72. doi: 10.1073/pnas.1118046108
28. Paolino, G.; Esposito, I.; Hong, SM.; Basturk, O.; Mattiolo, P.; Kaneko, T.; Veronese, N.; Scarpa, A.; Adsay, V.; Luchini, C. Intraductal tubulopapillary neoplasm (ITPN) of the pancreas: a distinct entity among pancreatic tumors. *Histopathology.* **2022**;81(3):297-309. doi: 10.1111/his.14698.
29. Basturk, O.; Berger, MF.; Yamaguchi, H.; Adsay, V.; Askan, G.; Bhanot, UK.; Zehir A.; Carneiro, F.; Hong, SM.; Zamboni, G.; Dikoglu, E.; Jobanputra, V.; Wrzeszczynski, KO.; Balci, S.; Allen, P.; Ikari, N.; Takeuchi, S.; Akagawa, H.; Kanno, A.; Shimosegawa, T.; Morikawa, T.; Motoi, F.; Unno, M.; Higuchi, R.; Yamamoto, M.; Shimizu, K.; Furukawa, T.; Klimstra, DS. Pancreatic intraductal tubulopapillary neoplasm is genetically distinct from intraductal papillary mucinous neoplasm and ductal adenocarcinoma. *Mod Pathol*. **2017**;30(12):1760-1772. doi: 10.1038/modpathol.2017.60.
30. Assarzadegan, N., Babaniamansour, S.; Shi, J. Updates in the Diagnosis of Intraductal Neoplasms of the Pancreas. *Front Physiol*. **2022**;13:856803. doi: 10.3389/fphys.2022.856803. Erratum in: Front Physiol. 2022 May 13;13:923917.
31. Basturk, O.; Chung, SM.; Hruban, RH.; Adsay, NV.; Askan, G.; Iacobuzio-Donahue, C.; Balci, S.; Zee, SY.; Memis, B.; Shia, J.; Klimstra, DS. Distinct pathways of pathogenesis of intraductal oncocytic papillary neoplasms and intraductal papillary mucinous neoplasms of the pancreas. *Virchows Arch*. **2016**;469(5):523-532. doi: 10.1007/s00428-016-2014-x.
32. Singhi, AD.; Wood, LD.; Parks, E.; Torbenson, MS.; Felsenstein, M.; Hruban, RH.; Nikiforova, MN.; Wald, AI.; Kaya, C.; Nikiforov, YE.; Favazza, L.; He, J.; McGrath, K.; Fasanella, KE.; Brand, RE.; Lennon, AM.; Furlan, A.; Dasyam ,AK.; Zureikat, AH.; Zeh, HJ.; Lee, K.; Bartlett, DL.; Slivka, A. Recurrent Rearrangements in PRKACA and PRKACB in Intraductal Oncocytic Papillary Neoplasms of the Pancreas and Bile Duct. *Gastroenterology*. **2020** Feb;158(3):573-582.e2. doi: 10.1053/j.gastro.2019.10.028.
33. Hruban, RH.; van Mansfeld, AD.; Offerhaus, GJ.; van Weering, DH.; Allison, DC.; Goodman, SN.; Kensler, TW.; Bose, KK.; Cameron, JL.; Bos, JL. K-ras oncogene activation in adenocarcinoma of the human pancreas. A study of 82 carcinomas using a combination of mutant-enriched polymerase chain reaction analysis and allele-specific oligonucleotide hybridization. *Am J Pathol.* **1993**;143:545–554
34. Buscail, L.; Bournet, B.; Cordelier, P. Role of oncogenic KRAS in the diagnosis, prognosis and treatment of pancreatic cancer. *Nat. Rev. Gastroenterol. Hepatol.* **2020**;17:153–168. doi: 10.1038/s41575-019-0245-4.
